# Effects of Low-Pressure Systems on Temperature, Humidity, Egg Production, and Feed Utilization Efficiency in Large-Scale Poultry Houses during Summer

**DOI:** 10.3390/ani14172554

**Published:** 2024-09-02

**Authors:** Haiqing Peng, Yang Wang, Zhihao Zhang, Wenxiang Qin, Baoming Li, Weichao Zheng, Peng Yin, Hao Zhu

**Affiliations:** 1 Department of Agricultural Structure and Environmental Engineering, College of Water Resources and Civil Engineering, China Agricultural University, Beijing 100083, China; phq@cau.edu.cn (H.P.); wangyang512@cau.edu.cn (Y.W.); bs20223090751@cau.edu.cn (Z.Z.); wenxiang@cau.edu.cn (W.Q.); weichaozheng@cau.edu.cn (W.Z.); yinpeng@cau.edu.cn (P.Y.); 2 Key Laboratory of Agricultural Engineering in Structure and Environment, Ministry of Agriculture and Rural Affairs, Beijing 100083, China; 3 Beijing Engineering Research Center on Animal Healthy Environment, Beijing 100083, China; 4 Guangdong Sun Daily Farm Ecological Food Co., Ltd., Jiangmen 529300, China; haozhu5211@gmail.com

**Keywords:** extreme weather, thermal environment, production performance, laying hen, feed intake

## Abstract

**Simple Summary:**

Low-pressure systems frequently occur during the summer in coastal areas and can be detrimental to commercial poultry houses. The effects of air temperature and humidity vary across different periods. Throughout the generation of low-pressure systems, landfall, and disappearance, air temperature and humidity changes can interfere with the housing environment. However, farmers often overlook the impact of low-pressure systems on large-scale poultry houses, which may impair the production of laying hens. This study investigated the effects of the different low-pressure systems on the housing environment and egg production in large-scale poultry houses in China. The results indicated that a single low-pressure system reduced the laying performance, whereas a sustained low-pressure system mitigated the impact on egg production. This study aimed to determine the effect of low-pressure systems on the environment and the laying performance of large-scale poultry houses during the summer and to provide a basis for farmers to adjust their environmental control strategies under extreme weather conditions.

**Abstract:**

Low-pressure systems (LPSs) are among the most critical weather systems, producing excessive precipitation that causes air temperatures to drop and rise considerably. Acute temperature changes directly affect poultry feed intake (FI) and laying performance. To explore the effects of LPSs on hens, the parameters of air temperature, relative humidity, egg production, and feed utilization efficiency were evaluated during different LPSs in three houses. Results indicated that about 2.8 ± 0.7 d, 2.4 ± 0.5 d, and 2.4 ± 0.5 d before the LPS landfall in houses 1, 2, and 3, respectively, the indoor air temperature started to decrease, with the average decreases being 1.7 °C ± 0.4 °C, 2.4 °C ± 0.6 °C, and 1.8 °C ± 0.4 °C, respectively. Significant differences were observed between different LPSs for reducing indoor air temperature (*p* < 0.05) in the three houses. In house 1, the egg production rates (EPRs) were decreased by 6.6% and 1.1% when LPSs 1 and 2 landed. The average egg weight (AEW) and FI during the LPS landfall were significantly higher than those before the LPS landfall (*p* < 0.01). Under successive LPSs landfall in the three houses, the EPRs initially reduced by 3.9%, 4.0%, and 0.5%, respectively, but the second LPS event increased the EPRs by 1.8%, 5.3%, and 1.0%, respectively. Furthermore, the LPS landfall increased the feed conversion ratio (FCR_e_) in the three houses, all above 2.00. In conclusion, LPSs can reduce heat stress, lower the EPRs, and lead to higher FI, FCR_e_, and AEW.

## 1. Introduction

Evidence for the increasing frequency of extreme weather events is abundant worldwide. Low-pressure systems (LPSs) are among the most critical weather systems frequently observed in inland and coastal areas during the summer [1]. LPSs often cause extreme changes in the atmospheric environment [2], severely damaging agricultural production [3]. The total damage Typhoon Ompong caused to crops and livestock in the Philippines was estimated at PHP 26.8 billion based on data from the Department of Agriculture [4]. The LPS that hit the Orissa state in 1999 caused massive losses to the economy of the state by killing thousands of livestock, particularly cows (19.04%), bullocks (2.78%), calves (4.07%), buffaloes (4.08%), sheep (12.70%), goats (8.65%), pigs (6.43%), and poultry (24.37%) [5]. Poultry was the most affected by the LPS. Similarly, Typhoon Morakot caused the most significant damage to poultry in Taiwan, with 6.11 million poultry deaths and losses of TWD 721.71 million [6].

Due to its massive economic consequences on agricultural production, the impact of LPSs on livestock and poultry production should be emphasized [7]. Throughout the process of LPS generation, landfall, and disappearance, it can decrease air temperature and increase air humidity during the summer. At the same time, it can bring the opposite effect. The impact on temperature and humidity varies during different LPS periods. Wang et al. (2023) [8] reported the differential effects of typhoons on sea surface temperature before and after the transit of LPSs. The relative humidity of the atmosphere also increases after the transit of LPSs [9]. A previous study by Kanada and Aiki (2024) [10] demonstrated that LPSs of different sizes and types affect temperature and humidity differently. The high- and low-pressure areas formed by the LPSs also affect the poultry house’s indoor and outdoor air temperatures [11,12]. Consequently, the changes in temperature and humidity during LPSs interfere with the regular feeding and production environment for laying hens.

Increased frequency and intensity of extreme weather events can affect animal health through various mechanisms [13]. Egg production rate (EPR) and feed conversion ratio (FCR_e_) are susceptible to temperature [14,15]. Most studies quantitatively described the association between different environmental temperatures and egg production characteristics [16,17]. However, the temperature changes caused by extreme weather are rapid and dramatic. During acute cold stress, laying hens need to sustain their basal metabolic rate, which leads to reduced laying energy and, consequently, decreased egg production rates. [18]. The connection between egg production and air temperature fluctuation can be established as a nonperiodic relationship with a time delay [19]. When the indoor temperature fluctuates, the feed intake (FI) changes [20], and egg production, egg weight, and the disease resistance of the hens also decreases [21]. Webster and Czarick (2000) [22] also found that egg production performance changes with temperature changes. Furthermore, Emmans and Charles (1989) [23] believed that temperature fluctuations affect the FI and, thus, the egg size. Temperature-related illness and metabolic changes pose a significant challenge for poultry [21]. However, breeders often overlook the impact of LPSs on large-scale poultry houses, which may impair layer production. Previous research has found a strong relationship between temperature, rainfall, relative humidity, egg production, and FI in poultry [24]. Obtaining sufficient data to discuss the relationship between LPSs, laying performance, and FI can provide measurements to adjust management practices in order to mitigate the impact of extreme weather on egg production.

This study aimed to determine the effect of the different periods of LPSs on the air temperature, relative humidity, temperature–humidity heat stress index (*THI*), EPR, average egg weight (AEW), FI, and FCR_e_ in large-scale poultry houses during summer.

## 2. Materials and Methods

### 2.1. Poultry Housing

**Figure 1:**
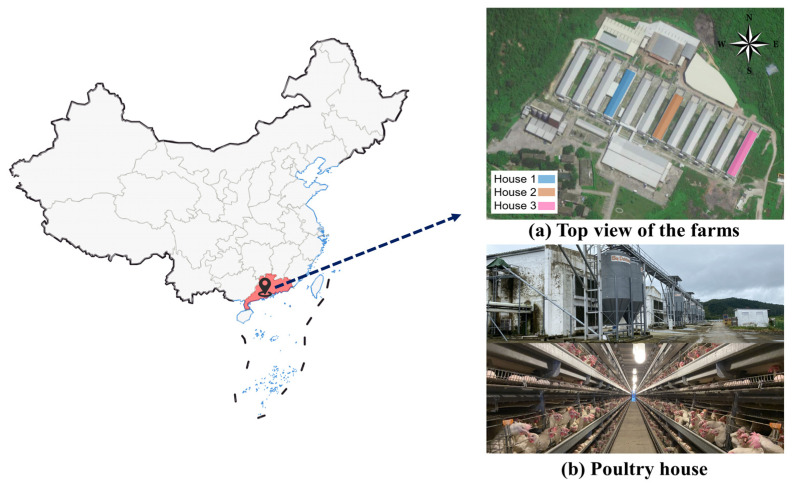
Experimental poultry house (Guangdong, China).

Data collection was conducted under the approval of the Institutional Animal Care and Use Committee of China Agricultural University. This experiment was conducted from 1 June to 31 August 2022 at a large-scale laying hen farm in Guangdong, China (Figure 1). Three houses were selected at random out of a total number of 12 houses. At the beginning of the experiments, 79,390, 85,132, and 81,531 Lohmann Pink laying hens aged 30, 18, and 16 wks were housed in houses 1, 2, and 3, respectively. The three houses had the same equipment configuration and were 87 m long, 14 m wide, and 7.5 m high. The poultry house had an eight-layer stacked cage system. In terms of layout, the inside of the house had five rows and six aisles, with each row having 124 cages. The stocking density of each cage is 49.4 pcs/m³. Feed was provided via feed conveyor chains, and manure was removed using a conveyor belt.

### 2.2. Ventilation and Environmental Control Mode

**Figure 2:**
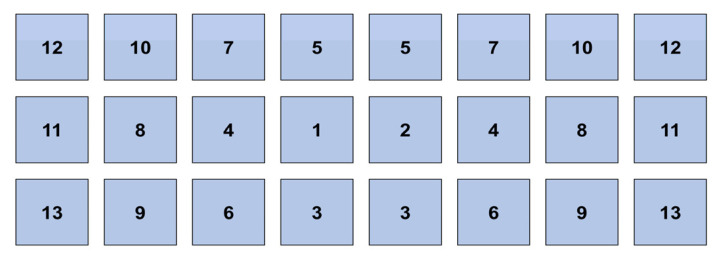
Ventilation level and fan position. Ventilation levels depend on the difference between the temperature in the house and the setting of the environmental controller. In the tunnel ventilation system, the initial indoor average air temperature is set at 21 °C, and for each degree Celsius increase, the ventilation level is increased by 1, up to a maximum of 13 levels.

The housing was equipped with tunnel ventilation and a wet-pad evaporative cooling system during the summer. Fresh air entered through the cooling pads and, subsequently, the inlets in both side walls, while exhaust air exited through the fans in one of the gable walls. The west gable wall had an evaporative cooling pad area of 56 m^2^, and the side wall had an evaporative cooling pad area of 2 × 72 m^2^. The wet curtain water pump began when the indoor average air temperature exceeded 29 °C and stopped when the indoor average air humidity exceeded 90%. The east gable wall was equipped with 24 fans with a diameter of 1.38 m and an exhaust volume of 41,000 m^3^/h. The number of fans turned on in the house was determined by the difference between the indoor and target temperatures, and 13 ventilation levels were set (Figure 2).

### 2.3. LPS Conditions

The LPS conditions were recorded from 1 June to 31 August 2022. The landfall time of an LPS is defined as the date when the surface circulation center of the LPS rises to land or a large island. The LPS landfall date was predicted about four days in advance based on warnings from the National Weather Service. Specific information on the LPSs, such as names and dates, were obtained from the National Weather Data Center. The local weather station provided rainfall data. Notably, two LPSs occurred on 4–10 August. These LPSs were considered consecutive LPSs to analyze the effect on egg production and feed utilization efficiency.

### 2.4. Temperature and Humidity on LPSs and Other Days

**Figure 3:**
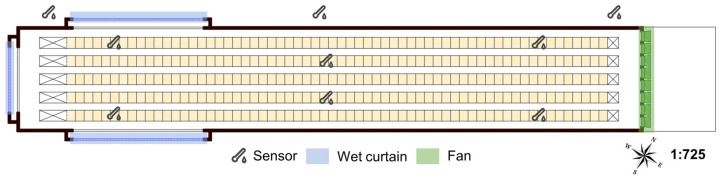
Testing points for temperature and relative humidity.

Portable temperature and humidity data loggers (Hobo U23-001, Onset Computer Corp., Bourne, MA, USA, accuracy ±0.2 °C and ±3%, respectively) were used to collect the three houses’ indoor and outdoor temperature data. The houses had the same sensor configuration. The data were recorded from 1 June to 31 August 2022. Data collection and storage were performed every 2 min. A schematic of the locations of the test sensors in the house is shown in Figure 3. There were 12 measurement points inside the house, with the same measurement points on the upper and lower levels. The measurement points were located at the hens’ breathing heights on the second and sixth floors of the poultry house. Three temperature sensors were installed outside the house to record changes in the external temperature.

### 2.5. Percentage of Weekly Time with Indoor Air Temperature above x °C

In this study, each collected datum point was treated as a constant temperature for 2 min to analyze the indoor average air temperature distribution inside the three houses on that day. The percentage of weekly time with indoor air temperature above *x* °C (*W_tx_*) is calculated as follows:

(1) $$W_{tx}=\frac{2n}{60\times 24\times 7}\times 100\%$$

where *x* denotes the values 27, 28, 29, and 30; *n* is the number of data points with temperatures above *x* °C in a day; and *W* is the percentage of weekly time with indoor air temperature time above *x* °C, %.

### 2.6. Temperature–Humidity Heat Stress Index (THI)

In this study, temperature and humidity are evaluated using the formula used by Wang et al. (2019) [25]. Thermal comfort in poultry is classified into four categories of heat stress based on *THI* values: Normal, less than or equal to 74; Alert, 75–78; Danger, 79–83; and Emergency, greater than or equal to 84 [25]. The *THI* is calculated as follows:

(2) $$THI=\left(1.8T_{db}+32\right)-\left[\left(0.55-0.0055RH\right)\times \left(1.8\times T_{db}-26\right)\right]$$

where *THI* is the temperature–humidity heat stress index for poultry; *T_db_* is the dry-bulb temperature of the air, °C; and *RH* is the relative humidity of the air, %.

### 2.7. Egg Production Condition

The commercial egg farm had an egg-grading machine (Omnia FT, 45,000–255,000 eggs/h, Moba Group, Gelderland, The Netherlands). The egg count and weight data were uploaded to the iMOBA data center with an accuracy of ±0.1% and ±1 g, respectively. iMOBA obtained these data daily from 1 June to 31 August 2022 in the three houses.

### 2.8. Feed Utilization Efficiency

All three houses had a feed tower and an environmental controller (Viper Touch, Big Dutchman, Vechta-Calveslage, Germany) connected to the feed tower weight sensor (FW99, Big Dutchman, Germany, accuracy ±0.1 kg). The FI values in the three houses were recorded daily from 00:00 to 24:00 from 1 June to 31 August 2022. The data were uploaded to the PC-side BigFarmNet Manager software 4.3. The feed conversion ratio (FCR_e_) is calculated as follows:

(3) $$FCR_{e=}\frac{\sum_{i}^{d}E_{i}}{\sum_{i}^{d}C_{i}}$$

where *E_i_* is the feed intake on day *i*, kg; *C_i_* is the egg mass on day *i*, kg; and *d* is the number of days in the test period.

### 2.9. Statistical Analysis

All data were tested using the Shapiro–Wilk test (for normal distribution) and the F test (for homogeneity of variances). Nonparametric statistics were used to analyze data with a non-normal distribution that transformation could not solve. All experimental data were obtained in the three houses from 1 June to 31 August 2022. Rainfall was calculated based on the total daily rainfall and analyzed and plotted. The temporal effects of the daily indoor and outdoor air temperature and humidity averages were evaluated based on the recorded LPS landfall dates. Unfortunately, some humidity data for house 2 was lost during the collection and processing. The values 27, 28, 29, and 30 were selected for *x* in *W_tx_* in houses 1, 2, and 3. *W_tx_* was calculated weekly. In addition, the AEW, EPR, FI, and FCR_e_ were recorded and analyzed in the three houses. Laying hens are at peak egg production until 33 weeks of age [26], so the effect of age on the laying performance was ignored. However, the laying hens in houses 2 and 3 were 18 and 16 weeks old, respectively. They had more significant variability in egg production and feed consumption. Therefore, the June and July data in houses 2 and 3 were not analyzed. The day of the LPS landing was considered the central day. A total of seven days, three days before and three days after the central day, were selected to evaluate and plot the data for that week. To ensure the data were reasonable, the egg production (total egg count/bird count/day), AEW, and FCR_e_ were calculated weekly. The FI (total feed consumption/bird count/day) was calculated daily. The *W_tx_*, AEW, and FI were analyzed through a one-way analysis of variance, and post hoc multiple comparisons tests were conducted using the least significant difference. The data were expressed as mean ± standard deviation, and analytical tests were performed with *p* < 0.05 as the significance level. The experimental data were organized using Excel 2016 and analyzed using IBM SPSS Statistics 25.0 (Armonk, NY, USA). Graphs were drawn using the Origin 2019 software.

## 3. Results

### 3.1. Effect of LPSs on Air Temperature and Relative Humidity

**Figure 4:**
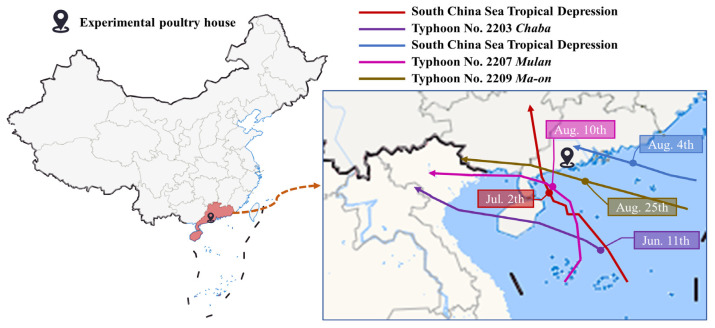
LPS landfall time, location, and LPS path. Boxes and circles represent LPS landfall time and location.

As shown in Figure 4, five LPSs occurred on 11 June (South China Sea Tropical Depression), 2 July (Typhoon No. 2203 Chaba), 4 August (South China Sea Tropical Depression), 10 August (Typhoon No. 2207 Mulan), and 25 August (Typhoon No. 2209 Ma-on), referred to as LPS 1 (L 1), LPS 2 (L 2), LPS 3 (L 3), LPS 4 (L 4), and LPS 5 (L 5), respectively.

**Figure 5:**
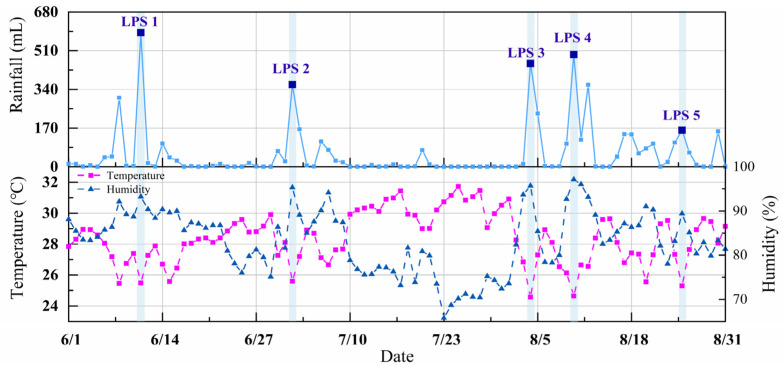
Effects of LPSs on air temperature and relative humidity.

The effects of LPSs on outdoor air temperature and relative humidity are shown in Figure 5. During the LPS, the air temperature and relative humidity varied between 24.6 °C and 26.7 °C and 89.4% and 97.1%, respectively; they also varied during the non-LPSs between 24.6 °C and 31.7 °C and 65.8% and 97.1%, respectively. Compared with the day before the LPS landfall, the temperature was up to 2.5 °C lower, and the humidity was up to 13.7% higher than during the LPS landfall. The day after the LPS landfall, the maximum increase in temperature was 2.7 °C, and the maximum decrease in humidity was 10.3%. The results indicate that LPSs can considerably reduce the air temperature and increase relative humidity (*p* < 0.05).

**Figure 6:**
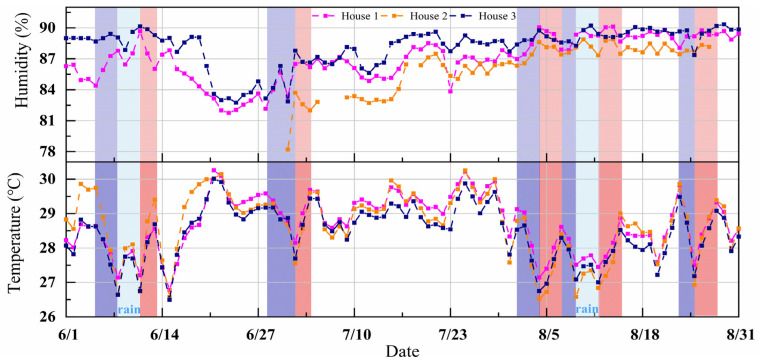
Effect of LPSs on the indoor air temperature and relative humidity. Each point is a daily average. The blue background means that the indoor air temperature has decreased during that time. Conversely, the red background means the indoor air temperature has increased during that time.

Figure 6 shows the results and analysis of variation in indoor air temperature and relative humidity in the three houses. The indoor air temperatures of the three houses were low during the LPS landfall, which were 26.8 °C, 26.5 °C, and 26.5 °C, respectively. However, relative humidity was less affected by the LPSs and remained high during summer, with averages of 86.9% ± 2.1%, 86.2% ± 2.3%, and 88.1% ± 1.9%, respectively. The results indicated that 2.8 ± 0.7 d, 2.4 ± 0.5 d, and 2.4 ± 0.5 d before the LPS landfall in houses 1, 2, and 3, respectively, the indoor air temperatures started to decrease, with the average decreases being 1.7 °C ± 0.4 °C, 2.4 °C ± 0.6 °C, and 1.8 °C ± 0.4 °C, respectively. The average days when the indoor air temperatures returned to the previous level were the same for all three houses, 2.6 ± 0.5 d. Moreover, the indoor air temperatures during this period in houses 1, 2, and 3 increased by 1.5 °C ± 0.2 °C, 2.2 °C ± 0.3 °C, and 1.7 °C ± 0.2 °C, respectively. In summary, the three houses’ indoor air temperature and relative humidity exhibited the same trend of change caused by the LPSs.

### 3.2. Effect of LPSs on W_t27_, W_t28_, W_t29_, and W_t30_

**Figure 7:**
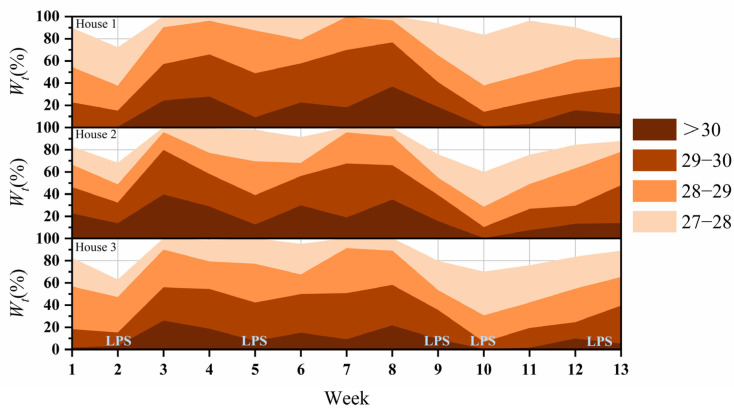
Effect of LPSs on *W_t27_*, *W_t28_*, *W_t29_*, and *W_t30_*.

The effects of the LPSs on *W_t27_*, *W_t28_*, *W_t29_*, and *W_t30_* in different weeks are shown in Figure 7. The changes in *W_t27_*, *W_t28_*, *W_t29_*, and *W_t30_* were synchronized. The maximum values of *W_t27_*, *W_t28_*, *W_t29_*, and *W_t30_* for the three houses were 100.0% ± 0.0%, 95.3% ± 3.4%, 71.2% ± 9.6%, and 33.8% ± 5.9%, respectively. At the time of a LPS landfall, the lowest values of *W_t27_*, *W_t28_*, *W_t29_*, and *W_t30_* in the three houses were 64.7% ± 5.3%, 32.0% ± 3.9%, 10.0% ± 3.0%, and 0.0% ± 0.0%, respectively. The successive LPS landfalls in weeks 9 and 10 caused a continuous decrease in the indoor air temperatures, with the average decreases in *W_t27_*, *W_t28_*, *W_t29_*, and *W_t30_* being 28.9% ± 9.4%, 60.1% ± 2.4%, 56.6% ± 4.6%, and 30.6% ± 6.6%, respectively. The decrease in *W_t27_*, *W_t28_*, *W_t29_*, and *W_t30_* in different LPSs is shown in Table 1. LPS 3 showed the greatest decrease in *W_t27_*, *W_t28_*, *W_t29_*, and *W_t30_*. Significant differences were observed between the different LPSs regarding decreases in *W_t27_*, *W_t28_*, *W_t29_*, and *W_t30_* (*p* < 0.05). Overall, LPSs reduce *W_t27_*, *W_t28_*, *W_t29_*, and *W_t30_* during summer.

### 3.3. Effect of LPSs on THI

The effects of LPSs on the *THI* are shown in Table 2. Due to missing data and to avoid the impact of continuous rainfall, LPS 5 was selected to analyze the effect of the *THI* in this result. The high summer temperatures and humidity in southern China’s coastal area cause severe heat stress. On 24 August, the emergency category was detected in three houses at 32.8%, 30.1%, and 29.2%. Compared with 24 August, the emergency category disappeared, and the heat stress of hens in the three houses was alleviated on 25 August (typhoon landfall). And the danger category in houses 1 and 3 was reduced by 4.4% and 16.6%. However, on 26 August (after the typhoon’s landfall), the danger category in the three houses increased by 20.1%, 16.9%, and 21.9%, respectively.

### 3.4. Effect of LPSs on the EPR and AEW

**Figure 8:**
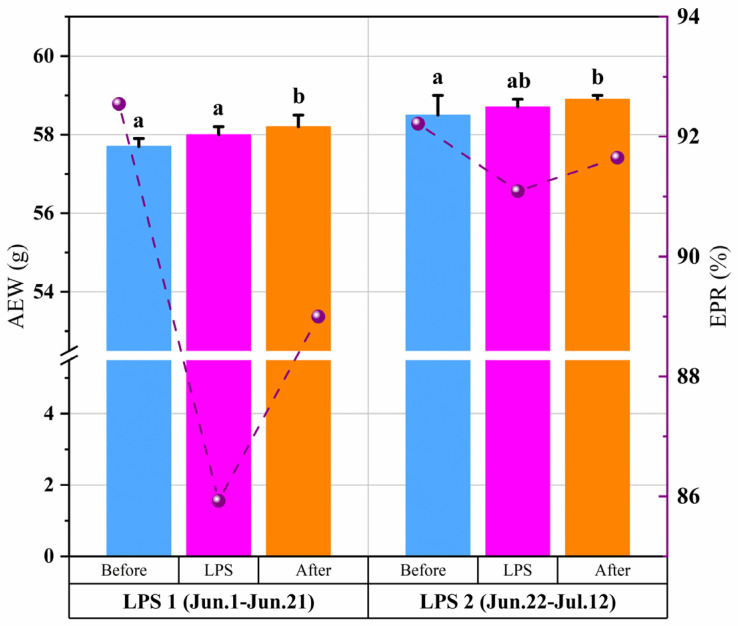
Effect of LPSs on egg production in house 1 (N = 79,015). The line graph represents EPR, and the bar graph represents AEW. a–b: Values without similar letters within the parameter are significantly different (*p* < 0.05).

The changes in the EPR during the different LPS periods are shown in Figure 8. The EPR and AEW trends were consistent under the influence of LPSs. During LPS 1 and 2, the EPR decreased by 6.6% and 1.1%, respectively. After the LPSs, the EPR gradually recovered to the pre-LPSs level. Meanwhile, a gradually increasing trend was observed in the AEW. After the LPSs, the AEW increased dramatically before the LPSs (*p* < 0.01). The highest AEW values were 58.2 ± 0.3 g and 58.9 ± 0.1 g during LPS 1 and 2, respectively. No significant difference was observed between the AEW values before and during the LPSs (*p* > 0.05).

**Figure 9:**
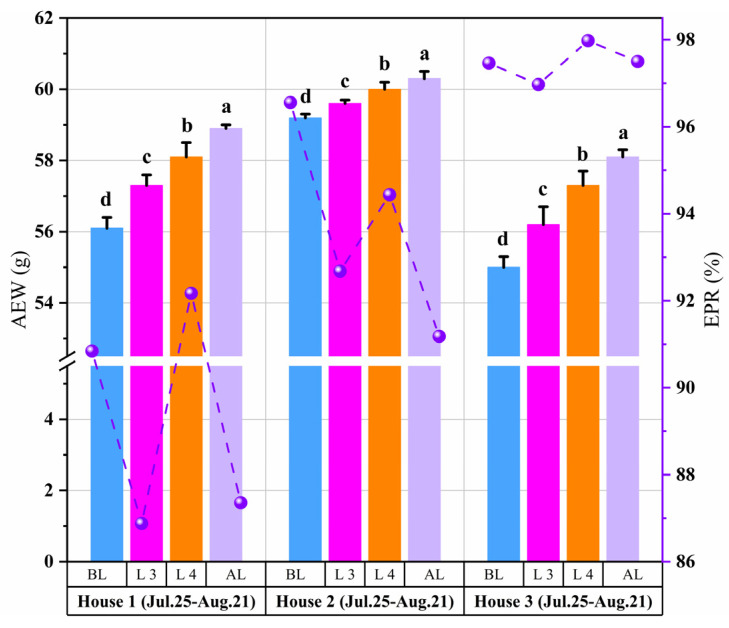
Effects of two consecutive LPSs on egg production in three houses. The sample sizes of hens in houses 1, 2, and 3 are 78,852, 84,618, and 80,429, respectively. The line graph represents EPR, and the bar graph represents AEW. a–d: Values without similar letters within the parameter are significantly different (*p* < 0.05).

The effects of two consecutive LPSs on egg production in LPSs 3 and 4 are shown in Figure 9. The three houses exhibited the same trend of changes, but the magnitude was different. The EPR of houses 1, 2, and 3 decreased by 3.9%, 4.0%, and 0.5%, respectively, during LPS 1. However, the EPR increased by 1.8%, 5.3%, and 1.0% during LPS 2. Under successive LPSs, the EPR initially decreased. However, the second LPS increased it. After the second LPS, the EPR decreased again. In house 3, the EPR was stable at 97% to 98%. In houses 1, 2, and 3, the AEW after the LPSs were 58.9 ± 0.1 g, 60.3 ± 0.2 g, and 58.1 ± 0.2 g, respectively, which were significantly higher than the AEW before the LPSs (*p* < 0.05). However, after the LPSs, the EPR still decreased and failed to recover to the pre-LPSs level.

### 3.5. Effect of LPSs on Feed Utilization Efficiency

The effects of LPSs on FI and FCR_e_ are shown in Table 3. The impact of different LPSs on FI is different. No significant difference was observed in FI between the LPS period and after the LPSs (*p* > 0.05) in LPS 1. Furthermore, the FI during the LPSs was significantly higher than before the LPSs (*p* < 0.01). The FI decreased after the LPSs. In addition, the LPS landfall increased the FCR_e_, which was above 2.00. After the LPSs, the FCR_e_ gradually returned to the pre-LPSs level.

The effects of two consecutive LPSs on the FI and FCR_e_ of the three houses are shown in Table 4. The trends of feed utilization efficiency were similar in different houses. The maximum FI value of the three houses was observed in LPS 4, with 112.9 ± 0.9, 115.8 ± 2.2, and 112.6 ± 2.8, respectively. The FI before the LPSs was significantly higher in three houses than during the LPS 4 period (*p* < 0.05). No significant difference was observed in the FI between the period of LPS 4 and after the LPSs (*p* > 0.05). Overall, the FI increased during the LPSs. Meanwhile, the FCR was increased by LPS 3 but was decreased by the subsequent landfall of LPS 4. After LPS 4 left, the FCR values of all three houses were higher than those before the LPSs.

## 4. Discussion

LPSs substantially impact temperature and humidity (Figure 5 and Figure 6). LPSs are often accompanied by extreme rainfall but have a negligible effect on the house’s humidity. This is because, in summer, the water pump is kept running to moisten the evaporative cooling pad to ensure ventilation and cooling, which results in high absolute humidity of the inlet air [27]. A substantial downward trend in house temperature was observed during the LPS landfall. Low air temperatures generally decrease the inlet air temperature, resulting in decreased indoor air temperature. It was also found that during winter and summer, the indoor air temperature of barns varies with their outdoor temperature [28]. In this study, LPS 5 increased the *W_t27_*, *W_t28_*, *W_t29_*, and *W_t30_*. However, as can be seen from Figure 6, the indoor air temperature decreased by 1.6 °C ± 0.2 °C in all three houses when LPS 5 landed. This may be due to the decrease in indoor air temperature from 16 to 21 August caused by persistent rainfall, which influenced the effects of LPS 5 on reducing the *W_t27_*, *W_t28_*, *W_t29_*, and *W_t30_* values. Different LPSs have different effects on temperature and humidity. The results are consistent with those of Kanada and Aiki (2024) [10], who also reported that large, slow- and fast-moving storms affect temperature differently. Differences in temperature and humidity were also observed in the three houses. The change in the indoor air temperature is related to the architectural structure and materials of the poultry house [29,30]. Although LPSs can reduce *W_t27_*, *W_t28_*, *W_t29_*, and *W_t30_*, they are ineffective on *W_t27_*. A possible explanation could be that the average outdoor temperature during the LPSs was 26 °C ± 0.5 °C, and the outdoor humidity was high, resulting in the poor cooling effect of the cooling pads [31].

In summary, the results indicated that LPS landfalls could reduce heat stress. High temperatures and humidity put hens under heat stress, and the *THI* should be of equal concern. Although lowering the *THI* under high temperatures is beneficial to laying hens, in conjunction with the study of egg production in this study, it appears that drastic changes in the *THI* do not have the desired effect on laying hen production. Kang et al. (2020) [32] suggest that an acute elevation of the *THI* has more severe effects on mortality in hens than gradual changes, even when temperature and humidity are similar in both cases. Humidity only affects hens when high temperatures are in effect [33]. This may be because the impact of the *THI* on egg production should be considered within the air velocity. The main weakness of this study was the lack of air velocity data. Increasing or decreasing the indoor air velocity has been determined to change the convective heat dissipation, adjust the perceived temperature, and alleviate stress [34,35]. Further work is needed to develop reliable analytical methods for the effects of ventilation and air velocity on the production and behavior of laying hens during LPSs.

Numerous studies have explored the effects of high temperature and humidity on egg production, including a decreased EPR [36]. The sudden drop in temperature caused by the LPSs should be seriously considered a health and production hazard for laying hens. As shown in Figure 8 and Figure 9, the marked drop in temperature induced by LPSs reduced the EPR in all houses. This finding is similar to that of the study by Campos et al. (2012) [37], which demonstrated that egg production was reduced in both breeds between the White Leghorns and heavy breeds during fast drops in ambient temperature. Acute temperature drops can reduce laying hens’ productivity and trigger diseases such as asthma and rhinitis [38,39]. Two consecutive LPSs effectively alleviated the high indoor air temperature, and the EPR increased in all three houses. These results are consistent with those of Oguntunji and Alabi (2010) [40], who found that if the indoor temperature changes relatively gently, hens can adapt to the changes in cold and hot environments within 3 to 5 days. And as shown in Figure 7, the *W_t30_* of house 3 is lower. This is why the EPR in house 3 stabilized at 97–98%. This agrees with the finding that laying hens have different high-temperature critical points and respond differently to heat stress [41,42]. However, after the LPSs, the EPR still decreased and failed to recover to the pre-LPSs level in houses 1 and 2, which is in agreement with the results of previous studies that found that cyclic heat stress negatively affected egg production and did not return to normal levels within four weeks [43]. In this study, the AEW increased during the consecutive LPSs, contrary to the results of Adams and Bell (1998) [44], who observed that the AEW is usually low during summer. However, in their study, only the AEW values of the four seasons were broadly compared, disregarding the effects of extreme summer weather. The other reason may be during the LPSs, the indoor air temperature decreased, and the AEW decreased. This result agrees with that of Rozenboim et al. (2011) [21], who found that laying hens exposed to 42 °C ± 3 °C for 15 d (heat stress) may have reduced egg weight compared with those exposed to 24 °C to 26 °C (thermoneutral conditions).

Laying hens “eat according to their ability”. In this study, we found that before the LPSs, the FI was significantly low when compared to the FI during and after the LPS. This result agrees with Perini et al. (2020) [45], who found that the FI substantially decreases during periods of higher temperatures and the water intake increases. A previous study reported that cooling can increase the maintenance energy consumption of laying hens [46]. In the present study, the LPSs alleviated the high indoor temperature conditions. They increased the FI, which is consistent with the results of Eltahan et al. (2023) [47], who found that increased heat dissipation improves FI in heat-stressed laying hens. It is generally believed that the heat stress response of laying hens only begins above 29 °C, but Mignon-Grasteau et al. (2015) [42] found that feed consumption decreases by 10% already at 24 °C. This explains why a decrease in FI was observed during the LPSs in our study. Furthermore, it was found that the FI substantially decreased under high-temperature cycling compared with that under thermoneutral temperature [48]. Star et al. (2008) [49] found that short- and long-term heat stresses have different effects on the feed consumption of laying hens and that laying hens have different adaptation mechanisms to these stresses. This study also proved this point. As shown in Table 4, laying hens of various ages exhibit different feed changes under consecutive LPSs. During the L3, the FI increases significantly compared to before the LPSs in house 1. House 1 had older laying hens and substantially higher FI than the other two houses. However, the changes in FI patterns before, during, and after the LPSs were the same for all houses throughout the consecutive LPSs. The results of our study, which focused on the fluctuation during the LPSs, align with previous research by Mignon-Grasteau et al. 2015 [42], who found that the FI of older hens was more easily affected by temperature fluctuation. When the ambient temperature suddenly increases, the hen’s body temperature rises, the metabolic rate increases, and the FI decreases [50].

Hens’ health and welfare status is closely linked to the weather [51]. LPSs are extreme weather events that are difficult to predict and monitor. Our study, which only tested data from three houses in one region for three months during summer, suggests more comprehensive research is needed. In particular, additional areas, egg production data, and environmental factors should be accumulated for further analysis, highlighting the importance of ongoing research and developing effective management strategies. Extreme weather events can significantly impact poultry, but the current impact on large-scale poultry operations needs to be better understood [4,5,6]. The amount of data is limited, but it can reveal specific patterns. According to Sunrise Farms’ 3 June 2024 feed prices [52], bulk non-GMO feed for laying hens is USD 625.0 per ton. About 2.4 tons of feed were added per LPS during the trial period in house 1. Every time an LPS landed, a poultry house with 80,000 hens lost USD 1500.0. In addition, according to the U.S. Bureau of Labor Statistics (BLS) data for April 2024, a dozen eggs cost USD 2.86. About 258 dozen eggs were lost per LPS during the trial period in house 1. So, it is worth noting that each LPS causes about USD 738.3 in damage to a poultry house with 80,000 hens [53]. In total, an LPS can cost large-scale egg farmers approximately USD 2238.3. Therefore, keeping an eye on the weather forecast during production is essential. For a single LPS, it is critical to manage indoor air humidity and lower air temperature before and after the LPS and safeguard the building’s airtightness during the LPS to reduce temperature loss. On the other hand, for consecutive LPS landfalls, it is significant to maintain the housing warmth after the first LPS and minimize the number of opening fans based on the actual indoor air temperature to avoid cold stress in hens due to prolonged cooling of the housing.

## 5. Conclusions

The results of this study indicated that the indoor air temperature started to decrease by 2.8 ± 0.7 d before the LPS landfall and increased within 2.6 ± 0.5 d after the LPS landfall. Significant differences were observed between the different LPSs regarding reduction of *W_t27_*, *W_t28_*, *W_t29_*, and *W_t30_* (*p* < 0.05). The LPSs can reduce the EPR, increase the FCR_e_, and substantially increase the AEW and FI. Furthermore, a persistent LPS can increase the EPR and decrease the FCR_e_. However, in this study, the EPR and FCR_e_ failed to return to the pre-LPSs level after the LPSs. This information helps us understand the impact of LPSs on the environment and egg production. Future studies should explore more management strategies for minimizing the effect of LPSs on farming.

## Figures and Tables

**Table 1:** Reduction of the percentage of weekly time with indoor air temperature above 27 °C (*W_t27_*), *W_t28_*, *W_t29_*, and *W_t30_* in different low-pressure systems (LPSs).

Period	LPS 1	LPS 2	LPS 3	LPS 4	LPS 5	*p* Value
*W_t27_*	−17.3% ± 2.0% ^a^	0.3% ± 1.3% ^c^	−16.9% ± 7.6% ^a^	−12.1% ± 2.8% ^ab^	−1.1% ± 7.9% ^bc^	0.015
*W_t28_*	−14.7% ± 3.6% ^c^	−6.1% ± 2.9% ^d^	−34.8% ± 2.4% ^a^	−25.3% ± 1.9% ^b^	9.2% ± 5.3% ^e^	0.000
*W_t29_*	−8.2% ± 4.5% ^b^	−16.1% ± 2.9% ^b^	−28.5% ± 5.6% ^a^	−28.1% ± 1.0% ^a^	13.1% ± 5.3% ^c^	0.000
*W_t30_*	−2.3% ± 4.6% ^b^	−15.3% ± 3.2% ^a^	−16.7% ± 3.1% ^a^	−13.9% ± 3.7% ^a^	−2.5% ± 2.3% ^b^	0.003

^a–e^: Values without similar letters within the parameter differ significantly (*p* < 0.05).

**Table 2:** Effect of low-pressure systems (LPSs) on *THI*. 25 August is the landfall date of Typhoon No. 2209 Ma-on.

	Date	THI	Normal	Alert	Danger	Emergency
House 1	24 August	82.7 ± 1.9	0.0%	0.0%	67.2%	32.8%
	25 August	79.6 ± 1.1	0.0%	36.2%	62.8%	0.0%
	26 August	81.5 ± 1.8	0.0%	16.7%	83.3%	0.0%
House 2	24 August	82.4 ± 2.0	0.0%	0.0%	69.9%	30.1%
	25 August	80.1 ± 1.0	0.0%	16.9%	83.1%	0.0%
	26 August	81.7 ± 1.6	0.0%	0.0%	100.0%	0.0%
House 3	24 August	82.3 ± 1.9	0.0%	0.0%	70.8%	29.2%
	25 August	79.3 ± 1.3	0.0%	45.8%	54.2%	0.0%
	26 August	81.1 ± 1.7	0.0%	24.9%	76.1%	0.0%

**Table 3:** Effects of low-pressure systems (LPSs) on the feed intake (FI) and feed conversion ratio (FCR_e_) in house 1 (N = 79,015).

Period	L 1			*p* Value	L 2			*p* Value
	Before	LPSs	After		Before	LPSs	After	
FI (g)	103.6 ± 3.4 ^b^	108.3 ± 1.8 ^a^	105.4 ± 3.3 ^ab^	0.025	104.9 ± 3.0 ^b^	108.9 ± 1.6 ^a^	105.9 ± 2.3 ^b^	0.015
FCR_e_	1.97	2.18	2.04	–	1.99	2.01	1.99	–

^a,b^: Values without similar letters within the parameter differ significantly (*p* < 0.05).

**Table 4:** Effects of two consecutive low-pressure systems (LPSs) on the feed intake (FI) and feed conversion ratio (FCR_e_). The sample sizes of hens in houses 1, 2, and 3 are 78,852, 84,618, and 80,429, respectively.

Period		Before	L 3	L 4	After	*p* Value
FI (g)	House 1	109.5 ± 2.2 ^c^	111.2 ± 0.8 ^b^	112.9 ± 0.9 ^a^	111.7 ± 1.2 ^a^	0.000
	House 2	106.5 ± 6.3 ^b^	111.4 ±2.7 ^ab^	115.8 ± 2.2 ^a^	110.9 ± 5.7 ^ab^	0.009
	House 3	105.3 ± 2.4 ^b^	109.2 ± 4.0 ^ab^	112.6 ±2.8 ^a^	108.8 ± 5.3 ^ab^	0.013
FCR_e_	House 1	2.05	2.15	2.06	2.16	–
	House 2	1.99	2.08	2.06	2.09	–
	House 3	1.90	2.05	2.01	1.96	–

^a–c^: Values without similar letters within the parameter are significantly different (*p* < 0.05).

## Data Availability

None were deposited in an official repository. The data that support the study findings are available upon request.
